# Identification and comparison of proteomic and peptide profiles of mung bean seeds and sprouts

**DOI:** 10.1186/s13065-020-00700-7

**Published:** 2020-07-29

**Authors:** Wei Yu, Guifang Zhang, Weihao Wang, Caixia Jiang, Longkui Cao

**Affiliations:** Heilongjiang Bayi Agricultural University National Coarse Cereals Engineering Research Center, Daqing, 163319 Heilongjiang China

**Keywords:** Mung bean seeds, Mung bean sprouts, Protein, Label-free proteomics, Peptide, Peptidomics

## Abstract

The objectives of this study were to analyze and compare the proteomic and peptide profiles of mung bean (*Vigna radiata*) seeds and sprouts. Label-free proteomics and peptidomics technologies allowed the identification and relative quantification of proteins and peptides. There were 1918 and 1955 proteins identified in mung bean seeds and sprouts, respectively. The most common biological process of proteins in these two samples was the metabolic process, followed by cellular process and single-organism process. Their dominant molecular functions were catalytic activity, binding, and structural molecule activity, and the majority of them were the cell, cell part, and organelle proteins. These proteins were primarily involved in metabolic pathways, biosynthesis of secondary metabolites, and ribosome. PCA and HCA results indicated the proteomic profile varied significantly during mung bean germination. A total of 260 differential proteins between mung bean seeds and sprouts were selected based on their relative abundance, which were associated with the specific metabolism during seed germination. There were 2364 peptides identified and 76 potential bioactive peptides screened based on the in silico analysis. Both the types and concentration of the peptides in mung bean sprouts were higher than those in seeds, and the content of bioactive peptides in mung bean sprouts was deduced to be higher.

## Introduction

The mung bean (*Vigna radiata*) has been widely consumed as one of the most valuable edible legume crop sources in many countries for a long time, such as China, Canada, and the United States [1]. The popularity of mung bean is related to its specific growth characteristics, such as relative drought tolerance and short growth cycle (70–90 days) [2]. Importantly, mung bean contains balanced nutrients and has a high nutritional value [3]. In particular, mung bean seeds are rich in protein (18–32%) and the mung bean protein is more digestible and less allergic than other legume proteins, which indicated it could be an excellent source of protein [4]. The majority of the mung bean proteins were mung bean storage proteins, which mainly consists of globulin and albumin. The compositions and content of mung bean proteins could influence the bioactivity and functionality of mung bean [5]. At present, mung bean proteins were mainly identified by two-dimensional electrophoresis combined with liquid chromatograph-mass spectrometer analyses [6, 7]. However, some limitations exist, low abundant, hydrophobic, extreme isoelectric point, and molecular weight proteins are rarely detected, preventing a complete description of the proteome [8]. Approximately 15% of mung bean proteins have not yet been extensively studied until now [1]. Recently, with the development of proteomic technique, label-free proteomics enables efficient and accurate identification and relative quantification of proteins, and it has been commonly used in plant proteomes research [9]. Plant peptides, whether they are extracellular or intracellular, have various physiological functions, such as signaling and defense [10]. Certain bioactivities including angiotensin I-converting enzyme (ACE) inhibitory activity, antioxidant activity, and antibacterial activity have been identified in the peptides of the mung bean protein hydrolysate [11]. To date, only three kinds of mung bean peptides (KDYRL, VTPALR, and KLPAGTLF) have been confirmed to have ACE inhibitory activity [12]. There are limited reports on the comprehensive proteomic and peptide profiles of mung bean.

Mung beans can be eaten cooked, fermented, or ground into flour. Also, mung beans are usually processed into soups or germinated into sprouts [13]. Mung bean sprouts have higher nutritional values and antioxidant activities compared to raw seeds [14]. They have the potential to prevent certain chronic diseases and cancers [15]. During germination, aerobic respiration and biochemical metabolism resulted in the hydrolysis of protein, carbohydrate, and fat in mung beans, as well as the formation of a series of metabolites [16]. The structural proteins are newly synthesized [17], and the mung bean storage proteins are degraded to new peptides, which influence the health claims of mung bean sprouts [13]. However, there is very limited data on the changes in proteins and peptides during the sprouting of mung bean.

Therefore, the objectives of this study were to analyze and compare the proteomic and peptide profiles of mung bean seeds and sprouts. Label-free proteomics and peptidomics technologies allow the identification and relative quantification of proteins and peptides in mung bean seeds and sprouts. Comparative studies on the proteomic and peptide profiles of mung bean seeds and sprouts contribute to clarify the impact of sprouting on the nutrition and function of mung beans. The results could promote a better understanding of the nutrition of mung bean seeds and sprouts, establish a fundamental study for further processing and application of mung beans, and provide comprehensive insights into the various mechanisms of germination in mung bean.

## Materials and methods

### Materials

Mung bean (*Vigna radiata*) was purchased from Shanxi Dongfangliang Life Science and Technology Co., Ltd (Datong, Shanxi, China), and stored at 4 °C. The mung bean sprouts were prepared as previously described [18], with some modifications. Mung bean seeds were soaked in excess water for 10 h at room temperature (22 ± 2 °C) and then drained. The soaked mung bean seeds were tiled into the germination tray, which then was placed in an artificial climate box to germinate for 5 days in darkness. The temperature and humidity were set at 22 °C and 80%, respectively. The mung bean sprouts were lyophilized for further analysis.

### Proteomic profiling analysis of mung bean seeds and sprouts

#### Extraction of mung bean protein

The mung bean proteins were extracted as described by Wiśniewski et al. [19], with some modifications. Mung bean seeds and sprouts were homogenized in lysis buffer consisting of 4% sodium dodecyl sulfate, 1 mM DL-Dithiothreitol, 150 mM Tris–HCl pH 8.0, and 1% protease inhibitor (Sigma-Aldrich, MO, USA). The homogenate was incubated in boiling water for 3 min and then sonicated on ice. The crude extract was incubated in boiling water again and centrifuged at 16,000×*g* at 25 °C for 10 min to collect the supernatants. Simultaneously, the BCA protein assay kit (Bio-Rad, USA) was used to determine the protein concentration.

#### Protein digestion

The protein was digested using the filter-aided sample preparation procedure, as previously described [19]. Briefly, 250 μg protein was mixed with 200 μL UA buffer (8 M Urea, 150 mM Tris–HCl pH 8.0) and centrifuged in a 10 kDa ultrafiltration tube at 14,000×*g* for 15 min. The precipitate was mixed with 100 μL 0.05 M iodoacetamide in UA buffer and incubated for 30 min at room temperature in darkness. The mixture was then centrifuged at 14,000×*g* for 10 min and the filter was centrifuged three times with 100 μL UA buffer and twice again using 100 μL 25 mM NH_4_HCO_3_. Finally, the protein suspension was digested with 3 μg trypsin (Promega) in 40 μL 25 mM NH_4_HCO_3_ at 37 °C overnight to obtain the peptide filter. The peptide concentration was determined using UV spectroscopy at 280 nm.

#### Liquid chromatography-electrospray ionization tandem mass spectrometry analysis (LC–ESI–MS/MS)

The peptide mixtures were desalted using C18 Cartridges (Empore™SPE Cartridges C18 (standard density), bed inner diameter 7 mm, volume 3 mL, Sigma-Aldrich, MO, USA) and concentrated by vacuum centrifugation, which was subsequently reconstituted in 40 μL of 0.1% (v/v) trifluoroacetic acid (TFA) solution. MS experiments were carried out on a Q Exactive mass spectrometer (Thermo Scientific) that was coupled to Easy nLC (Proxeon Biosystems, now Thermo Fisher Scientific) as previously described [20]. Peptides (5 μg) was loaded onto a C18 reversed-phase column (Thermo Scientific Easy Column, 10 cm long, 75 μm inner diameter, 3 μm resin) equilibrated with buffer A (2% acetonitrile and 0.1% formic acid) and separated with a linear gradient from 0–45% B (80% acetonitrile and 0.1% formic acid) for 105 min, followed by 45–100% B for 5 min and 100% B for 10 min, at the constant flow rate of 250 μL/min.

The data-dependent top10 method dynamically choosing the most abundant precursor ions from the survey scan (300–1800 m/z) for HCD fragmentation was applied to obtain the MS data. The target value was determined based on the predictive Automatic Gain Control. The dynamic exclusion duration was set as 25 s. Survey scans were acquired at a resolution of 70,000 at m/z 200 and resolution for HCD spectra was 17,500 at m/z 200. The normalized collision energy was 30 eV and the underfill ratio, which specifies the minimum percentage of the target value likely to be reached at maximum fill time, was defined as 0.1%. The instrument was run with peptide recognition mode enabled.

#### Sequence database searching and data analysis

The original MS data were analyzed using MaxQuant software version 1.3.0.5 and searched against the UniProt *Vigna radiata* database (35,454 total entries, 20191130). The search parameters were set as previously described [21]. and the label-free relative quantification was carried out in MaxQuant [22]. The abundance of protein was calculated based on the normalized spectral protein intensity (LFQ intensity). The UniProt-GOA database (http://www.ebi.ac.uk/GOA/) was used to annotate the gene ontology (GO) classification consisted of biological process, cellular component, and molecular function. Besides, the protein pathway was searched against the Kyoto encyclopedia of genes and genomes (KEGG) database (http://www.genome.jp/kegg/).

### Peptide profile analysis of mung bean seeds and sprouts

Peptides in mung bean seeds and sprouts were extracted as previously described with some modification [23]. The samples were quickly grounded in liquid nitrogen using a dry ice-cooled mortar and pestle, 5 g of bean powder was then dissolved in extraction buffer containing 1% TFA and 1% plant protease inhibitor (Sigma-Aldrich, MO, USA). Mixed samples were homogenized at 4 °C for 1 h and then sonicated with five short bursts of 6 s followed by intervals of 8 s for cooling on the ice. After that, samples were centrifuged at 10,000*g* for 20 min at 4 °C and filtered in an Amicon Ultracel 10 kDa Molecular weight (Mw) cut-off centrifuge filter tube (Millipore, MA, USA) by centrifugation (4 °C, 8000*g*) to remove peptides larger than 10 kDa. The desalting process, mass spectrometry conditions, and the data analysis were the same as the above proteomics analysis, except the peptide separation time was 60 min. Finally, the relative intensity of the peptide was obtained.

### Screening potential bioactive peptides

The potential bioactive peptides in mung beans were screened based on the in silico analysis [24]. The potential of the peptide to be biologically active was scored using the Peptide Ranker database (http://distilldeep.ucd.ie/PeptideRanker/). Good solubility is usually a prerequisite to exert the biological activity for peptides, therefore, the water solubility of the peptide is estimated using the Innovagen database (http://www.innovagen.com/proteomics-tools). The peptide with an instability index less than 40 is regarded as stable, and an instability index above 40 indicates the peptide may be unstable. Therefore, the ExPASy ProtParam tool (https://web.expasy.org/protparam/) was used to evaluate the stability of the peptide. In this study, peptides with scores > 0.5, good solubility, and instability index < 40 were regarded as the potential bioactive peptides [25].

### Statistical analysis

All experiments were carried out at least in triplicate. Independent-samples T-test was performed with the SPSS 19 version and the results were considered significant at *P* < 0.05. Principal component analysis (PCA) and hierarchical clustering analysis (HCA) were performed on SIMCA-P 14.1 (Umetrics AB, Umea, Sweden) and Multi experiment Viewer version 4.8 (www.tm4.org/mev/), respectively.

## Results and discussion

### Analysis of the proteomic profiles of mung bean seeds and sprouts

The protein concentration in mung bean sprouts (23.92 mg/mL) was lower than that in mung bean seeds (37.59 mg/mL), which could be due to the fact that the storage proteins were continuously hydrolyzed by the activated mung bean proteases to provide the necessary nutrition for seed germination and seedling growth [15]. It has been reported that the protein content of cowpea, jack bean, dolichos, and mucuna also decreased after germination [26]. A total of 2195 proteins were identified (Additional file 1: Table S1), which were significantly more than the mung bean proteome reported in previous studies [6, 27]. The types of proteins increased significantly after sprouting, which was in line with the result of Skylas et al. [27]. During germination, storage proteins are hydrolyzed and de novo synthesis of proteins occurs, which are both necessary for the completion of seed germination [28]. The variation of protein composition and content during germination reflects the balance between hydrolysis and synthesis of proteins. The major mung bean proteins, such as globulin, albumin, and glycinin, were all determined in this study. Globulin and albumin are the main mung bean storage proteins. The three types of globulins consisting of basic type (7S), vicilin type (8S) and legumin type (11S) globulins were all present in the mung bean seeds and sprouts. Moreover, the abundance of the globulins in mung bean seeds and sprouts accounted for 69.35% and 71.25% of the total protein abundance in the respective samples, which could be comparable with the results reported in the previous studies [1]. The greatest number proportion of these two samples was proteins with Mw between 20 and 40 kDa (Table 1), which could be related to the diversity of organelle proteins. For example, fifty-two of them were ribosomal proteins (40S, 60S, and 80S) involved in the formation of ribosomes and there were 43 mitochondrial proteins involved in mitochondrial function. However, the sum of the abundance of the proteins with Mw between 40 and 60 kDa accounted for more than 59% of the total protein abundance, due to the presence of globulins (subunits with molecular masses of 40–52 kDa).

**Table 1:** Statistics of molecular weights of identified proteins in mung bean seeds and sprouts

Mw range	Mung bean seeds			Mung bean sprouts		
(kDa)	Average number	Number percentage	Abundance percentage	Average number	Number percentage	Abundance percentage
0–20	278 ± 29	17.47	3.83	251 ± 37	16.47	4.31
20–40	528 ± 59	32.85	13.54	522 ± 78	33.09	11.99
40–60	432 ± 37	26.12	59.39	435 ± 36	26.24	60.83
60–80	169 ± 17	10.32	17.53	169 ± 10	10.48	17.62
80–100	104 ± 10	6.10	4.17	106 ± 6	6.14	4.01
>100	113 ± 10	7.14	1.54	120 ± 9	7.57	1.24

### GO and KEGG pathway analysis

**Fig. 1:**
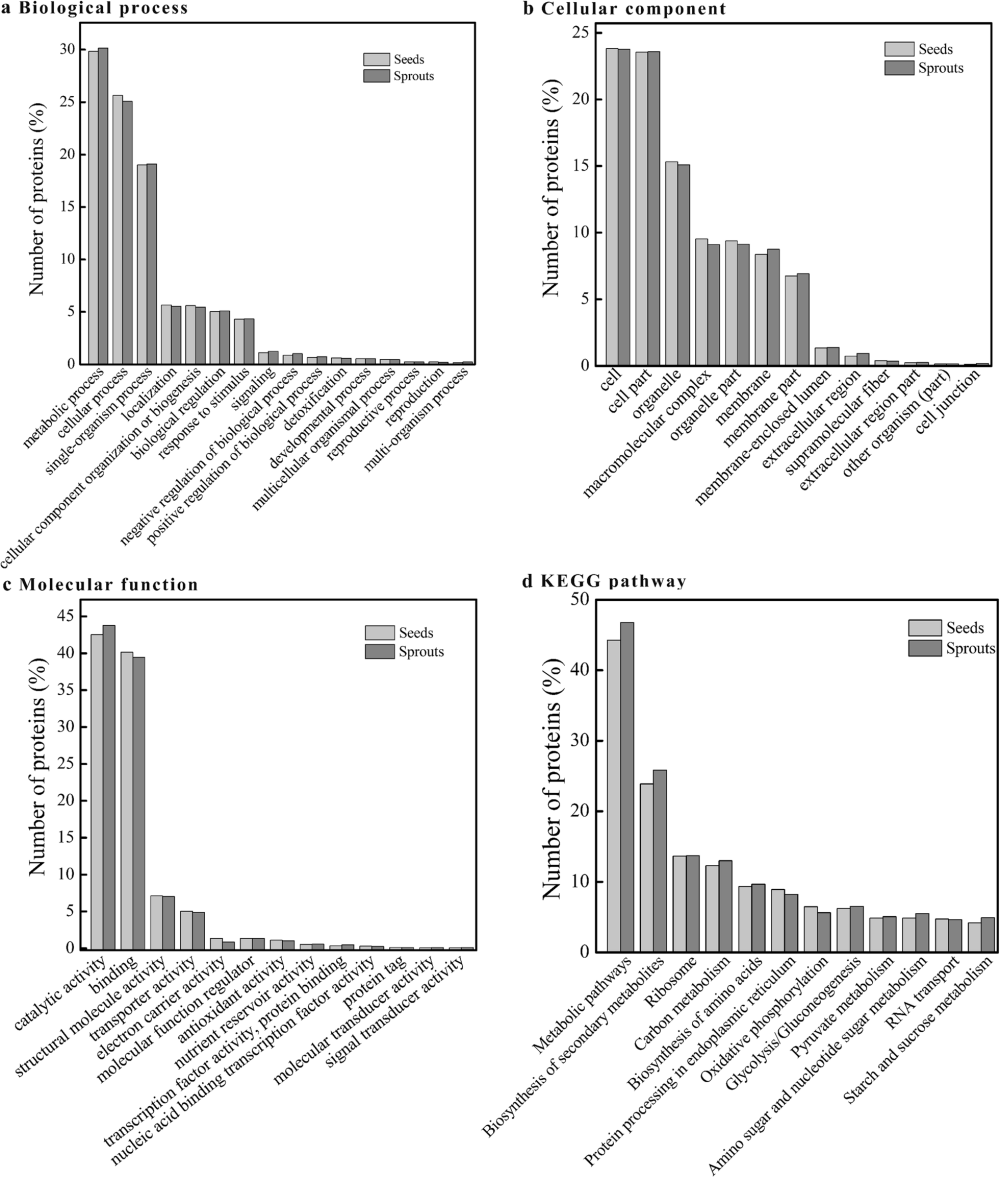
GO classification and KEGG pathways of proteins in mung bean seeds and sprouts

GO analysis is a good tool to explain the role of eukaryotic genes and proteins in cells, thereby comprehensively describing the properties of genes and gene products in organisms [29]. The exhaustive overview of the biological process, cellular component, and molecular function involved in all proteins is shown in Fig. 1. The most common biological process of proteins in mung bean seeds and sprouts was the metabolic process, followed by cellular process and single-organism process (Fig. 1a). Together they accounted for over 74% biological functions. A similar result for the key biological processes of germinating pea seed proteins has also been reported by Wang et al. [28]. Seed germination involves a complex coordination of various physiological, metabolic, and cellular processes, as previously described by Das et al. [30]. Dry legumes initially absorb water to activate a series of metabolic processes, accompanied by the reorganization of cellular structure. The top three predominant cellular components consisted of the cell, cell part, and organelle, and more than 62% of the identified proteins were located in them (Fig. 1b). The difference in the various molecular functions of the proteins was extremely obvious (Fig. 1c). The proportion of catalytic activity and binding activity was significantly higher than other molecular functions, which accounted for approximately 82% of all molecular functions. The highest catalytic activity might be related to the presence of large amounts of enzymes in plants. The KEGG pathways of all proteins identified in mung bean seeds and sprouts were analyzed (Fig. 1d). The results intuitively demonstrated that the majority of proteins in mung bean seeds and sprouts were involved in the metabolic pathways, followed by biosynthesis of secondary metabolites, ribosome, carbon metabolism, and biosynthesis of amino acids. Similar phenomena on molecular functions and KEGG pathways were observed in the rice proteins [31].

### PCA and HCA analysis

**Fig. 2:**
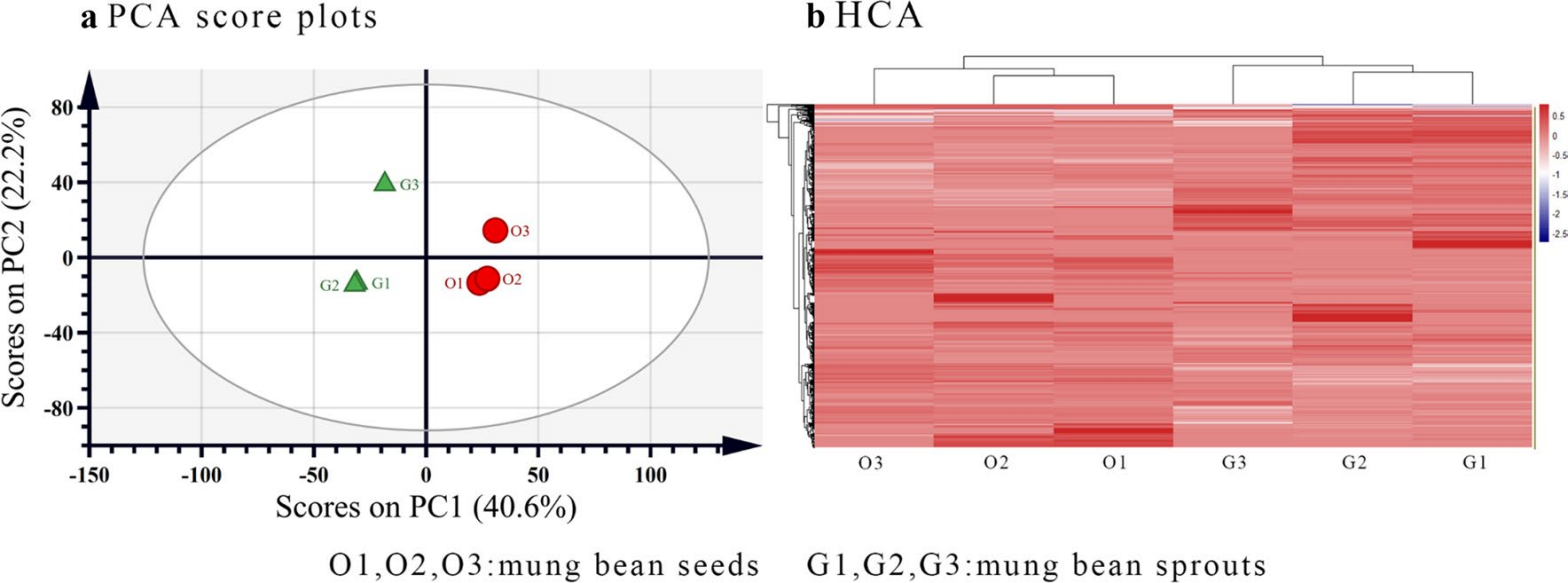
PCA and HCA analysis of proteomic profiles of mung bean seeds and sprouts

PCA was performed to visually distinguish the proteomic profiles of mung bean seeds and sprouts. There were three extracted principal components with a total variance of 79.9% (Fig. 2a), and the first two principal components (PC1 and PC2) separately accounted for 40.6% and 22.2% of the total variance, respectively. The proteomic profile of mung bean seeds was obviously separated from that of sprouts along PC1 (40.6% variance). To further evaluate the quantitative relationship of proteins among samples, heat-map visualization combined with hierarchical cluster analysis was utilized (Fig. 2b). It was evident that mung bean seeds and sprouts were separated into different clusters. All of the mung bean seed samples were grouped together in one cluster on the left side of the HCA dendrogram, while mung bean sprout samples were clustered on the right side. The normalized protein abundance was then visualised by colour: red–highest and blue–lowest values. Proteins in the right side of the heat map were omitted due to the limited graphics space, and which were consistent with the order of proteins in Table S1. Both the PCA and HCA results indicated that the proteomic profile varied significantly during mung bean germination.

### Analysis of differential proteins between mung bean seeds and sprouts

**Fig. 3:**
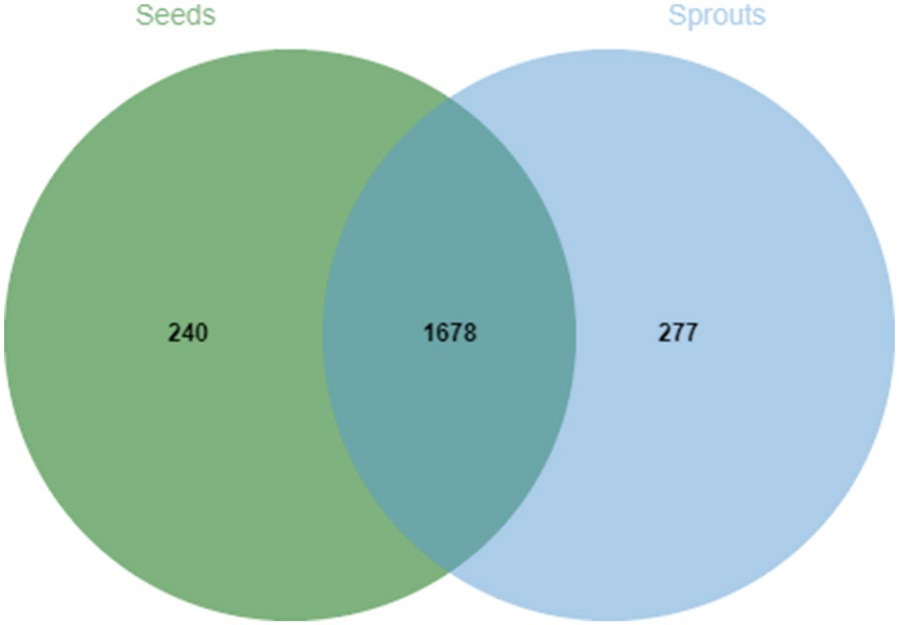
Venn diagram of the proteins identified in mung bean seeds and sprouts

**Fig. 4:**
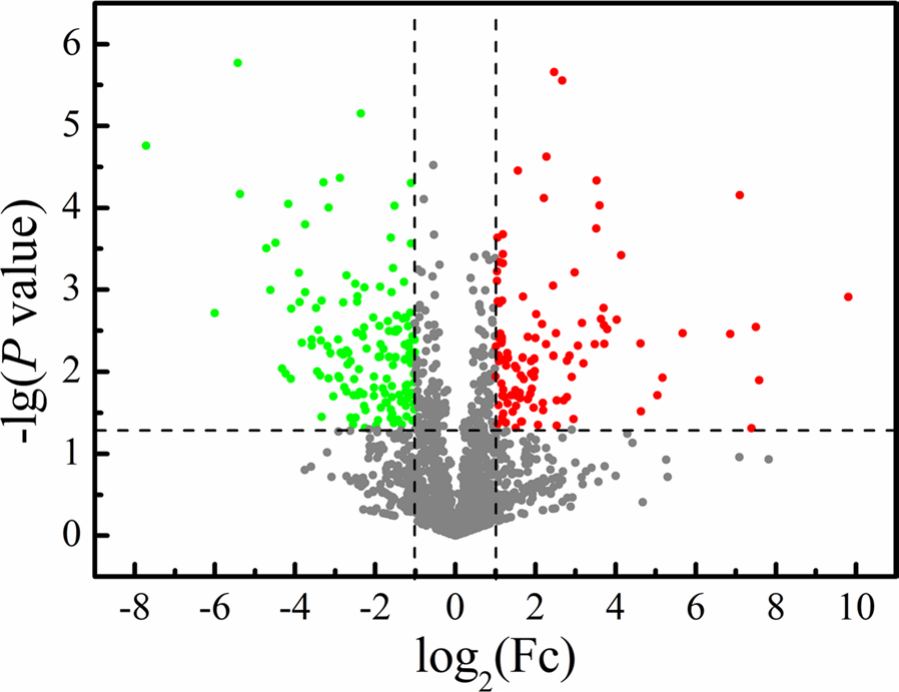
Volcano plot comparing proteomic profiles of mung bean seeds and sprouts

There were 240 and 277 proteins specific to mung bean seeds and sprouts, respectively. Moreover, there were 1678 consensus proteins identified in mung bean seeds and sprouts (Fig. 3). The differential abundance analysis of 1678 consensus proteins between mung bean seeds and sprout*s* was performed. Fold changes (Fc) were the specific values of the protein abundance in mung bean sprouts over that in mung bean seeds. Variables with Fc > 2 or < 0.5 and *P* < 0.05 were considered to be differential as previously described [21]. A volcano plot was then mapped to reflect the specific Fc and *P* value of each protein (Fig. 4). The green point with log_2_ (Fc) < − 1 and −lg (*P* value) > 1.301 represented the overabundant proteins in mung bean seeds, and the red point with log_2_ (Fc) > 1 and −lg (*P* value) > 1.301 represented the overabundant proteins in mung bean sprouts. Finally, 260 differential proteins were selected between mung bean seeds and sprouts. There were 149 proteins with higher abundance in the mung bean seeds and 111 proteins with higher abundance present in mung bean sprouts.

**Fig. 5:**
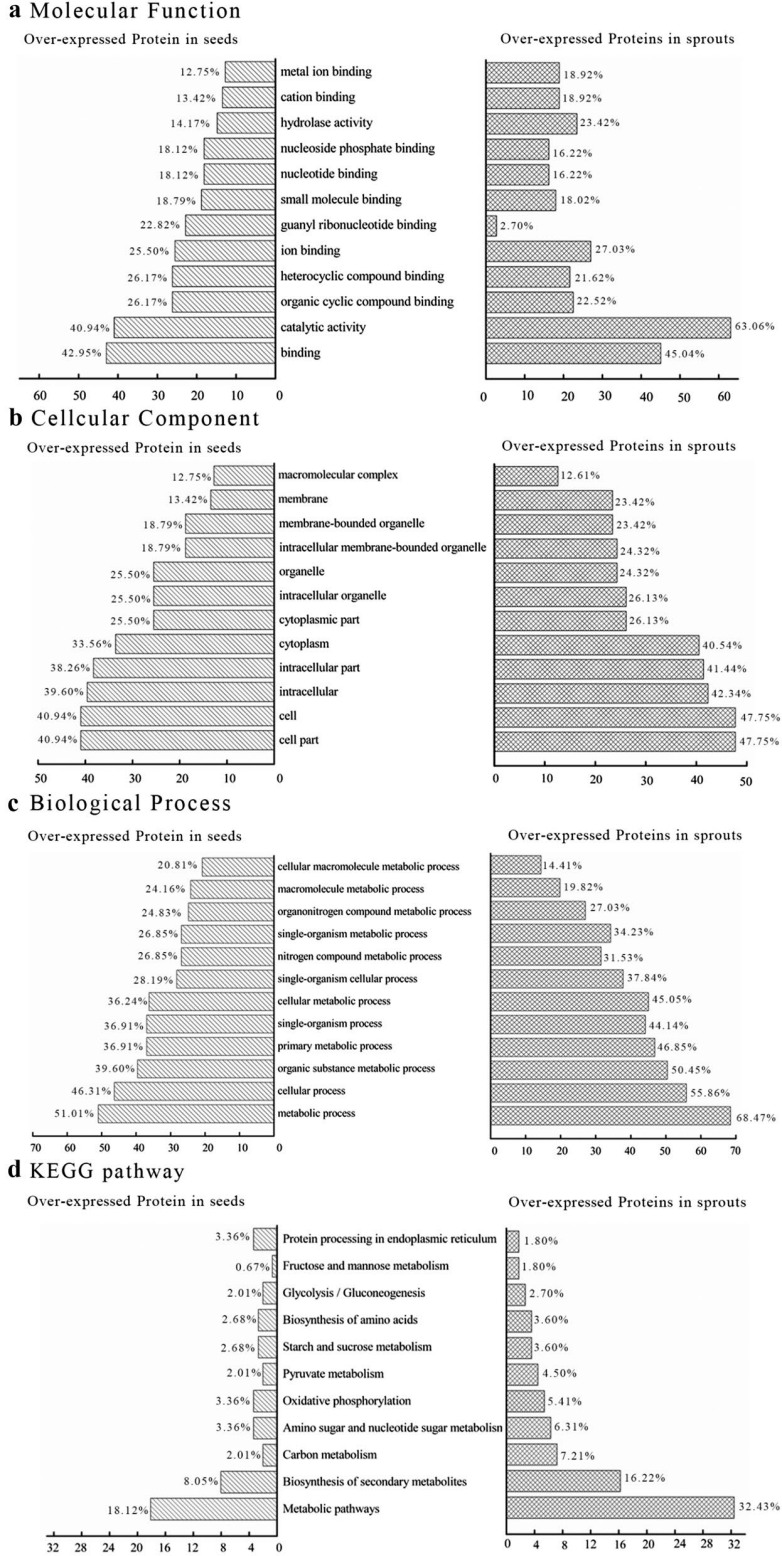
GO and KEGG analysis of differentially expressed proteins in the mung bean seeds and sprouts

The GO classification and KEGG pathway of these differentially expressed proteins were analyzed (Fig. 5). Binding (42.95–45.05%) and catalytic activity (40.94–63.06%) were the main molecular functions for the over-expressed proteins in both mung bean seeds and sprouts. More over-expressed proteins in mung bean seeds had guanyl ribonucleotide binding (22.82%) than those in sprouts (2.70%), while more over-expressed proteins in sprouts owned hydrolase activity (23.42%) compared with seeds (14.77%). Some guanyl ribonucleotide binding proteins, such as mitochondrial Rho GTPase and signal recognition particle receptor subunit beta, are GTPase-activating proteins (GAPs) [32]. GAPs enhance the hydrolysis of GTP during seed germination, thereby accelerating their inactivation, which explains why their abundance decreased significantly during germination [33]. Similarly, the abundance of ADP-ribosylation factor-like protein with guanyl ribonucleotide binding activity in seeds was obviously higher than that in sprouts, which might also be related to its capacity to bind and hydrolyse GTP [34]. The hydrolysis of storage reserves is one of the most important physiological processes during seed germination. Accumulated evidence suggests that the content of hydrolases of various plant seeds gradually increases during germination to induce the degradation of organic macromolecule into soluble substance for other tissues requirement, which is a conserved mechanism of seed germination [35]. Cellular component analysis indicated that the majority of differentially expressed proteins were cell part, cell, intracellular, and intracellular part proteins. The percentage of membrane proteins in the over-expressed proteins in mung bean sprouts (23.42%) was significantly higher than that in mung bean seeds (13.42%). The top three dominant biological processes consisted of metabolic process, cellular process, and organic substance metabolic process. Over-expressed proteins in mung bean seeds only had a more important role in macromolecule metabolic process (20.81%) and cellular macromolecule metabolic process (24.16%) than those in sprouts (14.41% and 19.82%, respectively). The majority of differentially expressed proteins belonged to the metabolic pathways, followed by carbohydrate metabolism and biosynthesis of secondary metabolites. More over-expressed proteins in mung bean seeds (3.36%) were involved in the protein processing in endoplasmic reticulum compared with sprouts (1.80%).

To investigate the individual differential proteins between mung bean seeds and sprouts, the 10 most over-expressed and 10 most under-expressed proteins in mung bean seeds/sprouts are shown in Table 2. The abundance of late embryogenesis abundant (LEA) protein D-34 isoform X2, LEA protein isoform X2, and LEA protein D-34 in mung bean sprouts were 0.005%, 0.024% and 0.038% of the corresponding protein abundance in mung bean seeds. This group of proteins accumulates in seed embryos and protects against water stress and seed dehydration through protein–protein interactions [27]. The abundance of this group of protein significantly decreased during germination due to the loss of seed embryo tissue. Seed biotin-containing protein SBP65 and seed biotin-containing protein SBP65-like were other proteins found to be more abundant in mung bean seeds than the sprouts. It has been reported that this kind of protein in pea had some similarities with LEA protein, including the amino acid compositions and hydrophilic characteristics [36]. During the process of germination, the dehydration tolerance was gradually lost, seed biotin-containing protein SBP65 as the stress response protein, was able to minimize the influence of the loss of dehydration tolerance on seed sprouting [28]. The abundance of lipoxygenases (LOXs) in mung bean sprouts was higher than that in mung bean seeds. LOXs were the key enzymes that promoted seed development during sprouting. The mung bean LOXs had similar biophysical and chemical characteristics to other legumes LOXs [37]. During the germination process, the LOXs were synthesized to degrade the lipid bodies in seeds [38]. The significant increase in the abundance of this type of protein during mung bean germination was consistent with the changes in this kind of protein during soybean and rice germination [39].

**Table 2:** Annotated results of the major differentially expressed proteins

Protein IDs	Protein names	Mw (kDa)	Length	Fc^a^	*P* value
A0A3Q0F8J7	Late embryogenesis abundant protein D-34 isoform X2	26.71	257	0.005	0.000
A0A1S3VUA0	Seed biotin-containing protein SBP65	50.18	476	0.016	0.002
A0A1S3VCS6	Protein SLE2	10.87	99	0.023	0.000
A0A3Q0EN85	Late embryogenesis abundant protein isoform X2	16.02	139	0.024	0.000
A0A1S3W001	Late embryogenesis abundant protein D-34	26.17	257	0.038	0.000
A0A1S3W170	Succinate dehydrogenase subunit 7B, mitochondrial isoform X2	10.79	94	0.041	0.001
A0A1S3W2J6	Seed biotin-containing protein SBP65-like	37.93	336	0.044	0.000
A0A1S3V296	Glycine cleavage system H protein	16.82	154	0.050	0.009
A0A1S3UI83	Formate dehydrogenase, mitochondrial (FDH) (EC 1.17.1.9)	41.80	381	0.053	0.010
A0A3Q0FEY5	Alpha-1,4 glucan phosphorylase (EC 2.4.1.1)	108.92	965	0.056	0.000
S5XAM1	Lipoxygenase (EC 1.13.11.-)	97.42	867	900.3	0.001
A0A1S3VKN3	Spermidine hydroxycinnamoyl transferase	50.24	449	191.3	0.013
A0A1S3U565	Lipoxygenase (EC 1.13.11.-)	96.64	856	181.1	0.003
A0A1S3V2R7	Uncharacterized protein LOC106771214	19.67	174	168.2	0.048
A0A1S3UVK8	1-deoxy-D-xylulose 5-phosphate reductoisomerase, chloroplastic	51.17	471	137.1	0.000
A0A1S3T7I3	Glucan endo-1,3-beta-glucosidase	37.60	342	116.3	0.003
A0A1S3V5U8	Phytochrome	123.96	1123	50.9	0.003
A0A1S3TVT1	4-hydroxy-3-methylbut-2-en-1-yl diphosphate synthase (Ferredoxin)	82.23	740	36.0	0.012
A0A1S3UU09	Pyruvate kinase (EC 2.7.1.40)	54.26	501	32.9	0.019
A0A1S3TQ18	DEAD-box ATP-dependent RNA helicase 3, chloroplastic isoform X2	84.65	776	24.8	0.030

^a^ Fc was the specific value of the protein abundance in mung bean sprouts over that in mung bean seeds

### The peptide profiles of mung bean seeds and sprouts

**Fig. 6:**
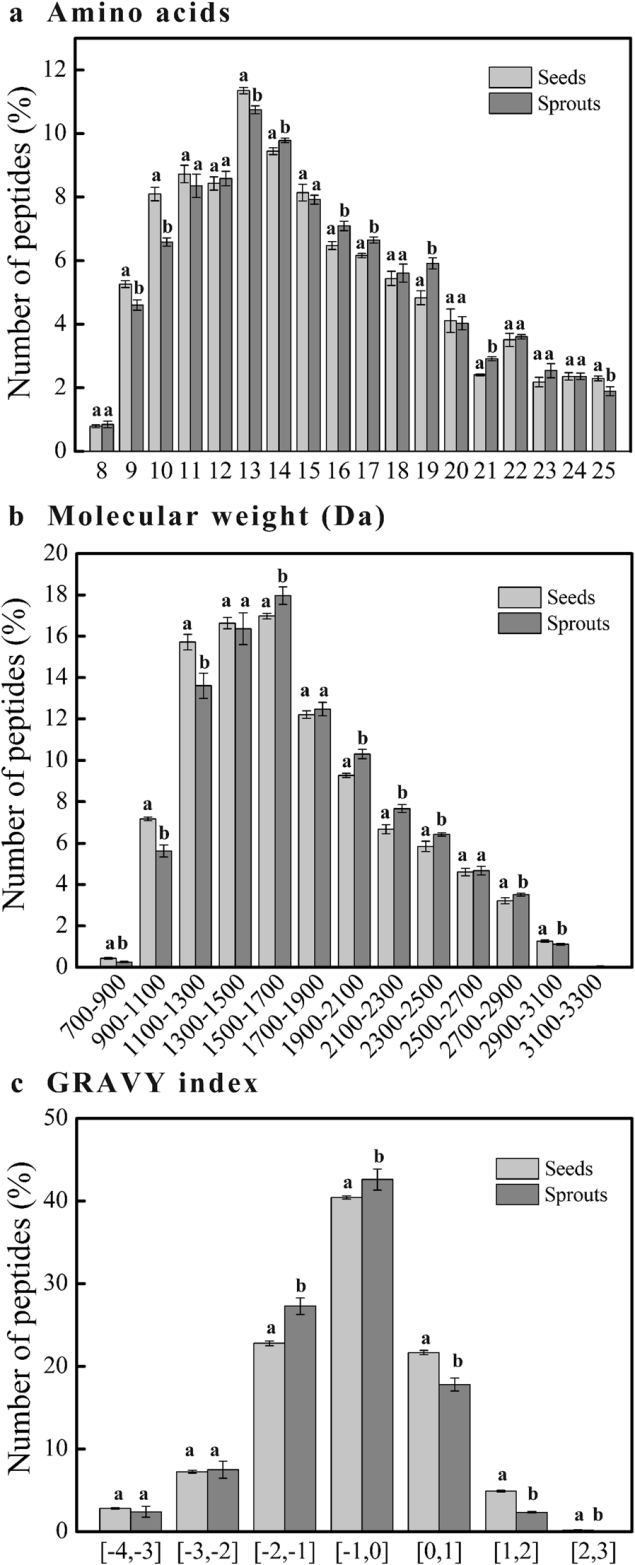
Physicochemical properties of the peptides identified in mung bean seeds and sprouts. Bars with different letters for each abscissa differ significantly (*P* < 0.05)

A total of 2364 peptides were identified and the detailed information is shown in additional file 2: Table S2. As far as we are aware, no studies have been previously published on the peptide profiles of mung bean seeds and sprouts using a peptidomics approach. There were 1662 and 1795 peptides present in mung bean seeds and sprouts, respectively. The number of peptides increased after sprouting, because the storage proteins were hydrolyzed into peptides under the action of the activated mung bean proteases [15]. The sequences of the identified peptides ranged from 8 to 25 amino acids, the Mw of peptides varied from 762.39 Da to 3241.55 Da and their grand average of hydropathicity (GRAVY) indexes distributed from −3.64 to 2.518. These physicochemical properties were distributed across a relatively wide span, reflecting the peptides identified have a large physicochemical diversity [40], which further indicates that the peptides in mung bean seeds and sprouts were effectively identified. The detailed distribution of the basic physicochemical characteristics of the peptides is shown in Fig. 6. Usually, peptides that consisted of less than 20 amino acids are prone to be absorbed [41], and there was no difference in the proportion of these peptides between mung bean seeds and sprouts (Fig. 6a). The peptides with Mw below 1300 Da in mung bean sprouts were less than those in mung bean seeds (Fig. 6b), which might result from the depletion of small peptides for de novo synthesis of proteins during germination. The peptides with the GRAVY index greater than 0 in mung bean seeds were more than those in mung bean sprouts, and vice versa (Fig. 6c), which indicated that there were more hydrophilic peptides in mung bean sprouts compared with seeds [42]. The hydrophobicity of peptide influences its absorption and bioactivity, therefore, the potential of peptides to exert their nutrition and functionality increased during mung bean germination. The peptides in mung bean seeds were derived from 180 proteins, while those in mung bean sprouts were released from 160 proteins. The occurrence might be related to the hydrolysis of certain peptides into undetectable small peptides during germination. The majority of the peptides in these two samples were originated from beta-conglycinin, beta chain-like (15.97% and 16.44%), followed by glycinin G4 (13.24% and 15.65%). These proteins are typical 7S and 11S storage globulins [17, 43], with relatively lower abundance compared with 8S globulins. The result indicated the number of peptides in the sample was independent of the abundance of the parent protein.

**Fig. 7:**
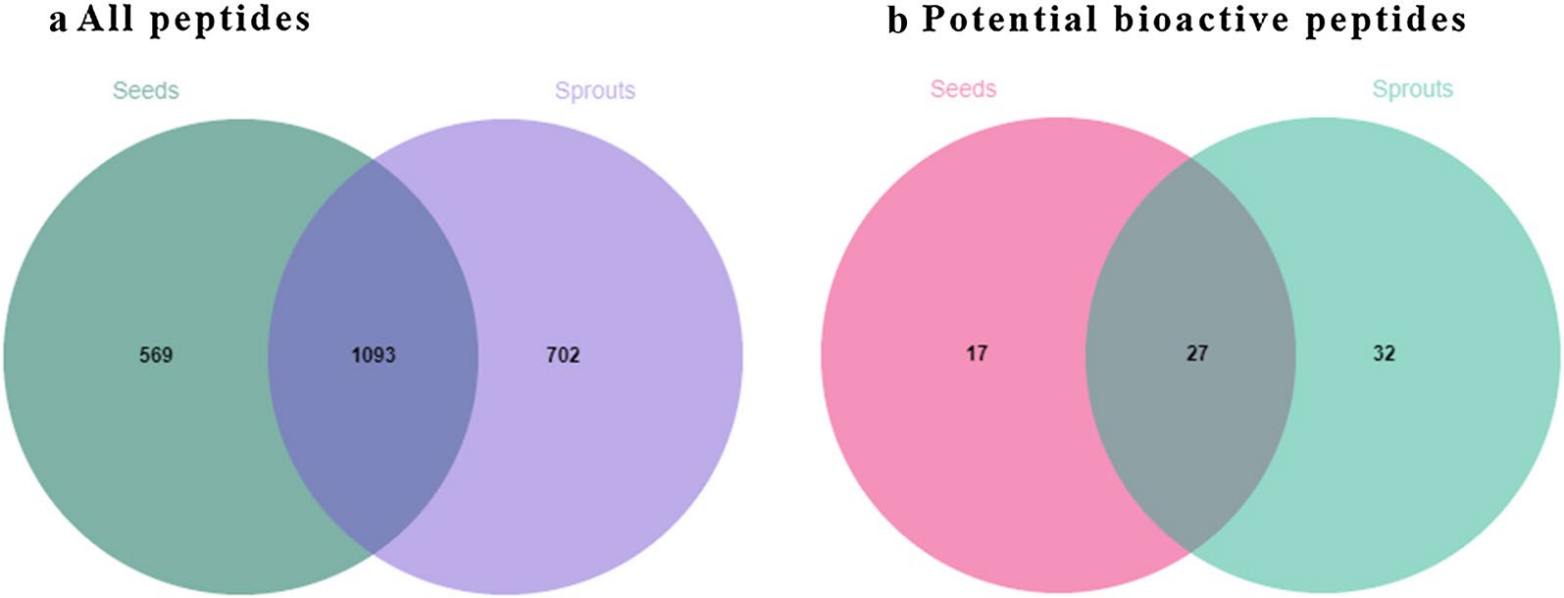
Venn diagram of peptides and potential bioactive peptides in mung bean seeds and sprouts

A Venn diagram of the peptides determined in the mung bean seeds and sprouts is shown in Fig. 7a. There were 1093 consensus peptides present in these two samples. Besides, there were 569 and 702 peptides specific to mung bean seeds and sprouts, respectively, which intuitively reflected the significant impact of germination on the peptide profile of mung beans. A total of 76 potential bioactive peptides screened is shown in Additional file 3: Table S3. There were 44 and 59 potential bioactive peptides present in mung bean seeds and sprouts, respectively. Their potential bioactive peptides accounted for 2.38% and 2.25% of the total peptide intensity in the respective samples. The peptide concentration in mung bean seeds and sprouts was 2.64 and 4.53 mg/mL, respectively. Therefore, we deduced that there were more bioactive peptides in mung bean sprouts than mung bean seeds, which could explain the more obvious biological activity of the mung bean sprouts after germination [13]. There were 27 consensus potential bioactive peptides present in these two samples, and there were 17 and 32 potential bioactive peptides specific to mung bean seeds and sprouts, respectively (Fig. 7b). Although the bioactivities of these peptides have not been confirmed, they provide directions for the screening and identification of mung bean bioactive peptides.

## Conclusion

In this study, quantitative proteomic and peptidomic analyses have provided novel molecular insights into the proteomic and peptidomic patterns of mung bean seeds and sprouts. Quantitative evidence revealed 111 proteins upregulated and 149 proteins downregulated during mung bean seed germination. Bioinformatics analysis indicated that the majority of them belonged to the cell part, cell, and intracellular compartments with binding and catalytic activities, which primarily involved in the metabolic process and cellular process. The results further confirmed that seed germination is mainly accompanied by the activation of metabolic processes and the reorganization of cellular structure. Several proteins, especially the LEA protein family and biotin-containing proteins, decreased significantly during germination, which was associated with their prevention of water stress and seed dehydration. Both the types and concentration of peptides increased after germination. Seventy-six potential bioactive peptides were screened based on the in silico analysis, and the content of bioactive peptides in mung bean sprouts was deduced to be higher than that in mung bean seeds. The proteomic and peptide profiles obtained in this study could promote a better understanding of the nutrition of mung bean seeds and sprouts, and provide comprehensive insights into the various mechanisms of germination in mung bean. Further researches will be required to confirm the bioactivities of these potential peptides in vitro and in vivo.

## Supplementary information

**Additional file 1: Table S1.** Identified proteins in the mung bean seeds and sprouts.

**Additional file 2: Table S2.** Identified peptides in the mung bean seeds and sprouts.

**Additional file 3: Table S3.** The potential bioactive peptides in the mung bean seeds and sprouts.

## Data Availability

The datasets generated during and/or analysed during the current study are available from the corresponding author on reasonable request.

## Abbreviations

ACE: Angiotensin I-converting enzyme
LC–ESI–MS/MS: Liquid chromatography-electrospray ionization tandem mass spectrometry analysis
TFA: Trifluoroacetic acid
GO: Gene ontology
KEGG: Kyoto encyclopedia of genes and genomes
PCA: Principal component analysis
HCA: Hierarchical clustering analysis
Mw: Molecular weight
Fc: Fold changes
GAPs: GTPase-activating proteins
LEA: Late embryogenesis abundant
LOXs: Lipoxygenases
GRAVY: Grand average of hydropathicity
